# Inflammatory Demyelinating Central Nervous System Diseases in Childhood: Clinical and Paraclinical Profiles in 133 Patients

**DOI:** 10.1155/2012/957802

**Published:** 2012-12-30

**Authors:** Derya Kaya, Egemen İdiman, Serkan Özakbaş

**Affiliations:** ^1^Department of Neurology, Ordu State Hospital, 52200 Ordu, Turkey; ^2^Department of Neurology, Faculty of Medicine, Dokuz Eylul University, 35340 Izmir, Turkey

## Abstract

In a retrospective review of patients with acquired demyelinating disorders of the central nervous system, 133 patients (5.6%) whose diseases started in childhood, were selected from 2369 patients, who had medical records in the Neurology Department of Dokuz Eylul University. Out of 133, 98 had relapsing remitting multiple sclerosis, 21 had secondary progressive multiple sclerosis, 8 had clinically isolated syndrome, 3 had neuromyelitis optica, 2 had Marburg disease, and 1 had radiologically isolated syndrome. In 55 patients (41.3%), disease onset was before age 16. Polysymptomatic presentation (22.6%) was the most common initial feature. The EDSS scores ranged from 0 to 9 with a median of 2.0 (2.22 ± 1.88) for 126 patients. MRI records of 111 patients were obtained. 97 patients had clinically definite multiple sclerosis. 11 MS patients (11.3%) did not initially present the diagnostic MRI features. All of the remaining multiple sclerosis patients fulfilled Barkhof-Tintore criteria (100%) and 88.7% fulfilled KIDMUS criteria. Cranial MRI of NMO patients was normal. Our findings demonstrate some important clinical and paraclinical features that can help the literature on acquired demyelinating disorders of childhood by utilizing data from Western Turkey.

## 1. Introduction

It was only in the late 1950s that multiple sclerosis (MS) in the pediatric population resurfaced in the literature [1, 2]. The latter half of the twentieth century led to a greater understanding of acquired central nervous system (CNS) demyelinating disorders [3]. The term “acquired demyelinating diseases” (ADD) applies more broadly to encompass the entire spectrum of acute demyelination. Clinically isolated syndrome (CIS) is often used to refer to the first attack of demyelination, especially to identify patients with high risk for MS [4]. The diagnosis of MS in children requires clinical (two or more attacks of neurologic impairment involving distinct areas of the CNS separated by more than 28 days [5]) or radiologic evidence of relapsing white matter disease (new T2 or gadolinium enhancing lesions more than three months after the initial episode) [6]. Neuromyelitis optica (NMO) is based on monophasic or multiphasic relapsing attacks of optic neuritis (ON) and/or acute transvers myelitis (ATM) [7]. These definitions are mainly clinical and include the differential of an initial demyelinating event, defined as a first acute or subacute clinical event with presumed inflammatory or demyelinating cause (monophasic acute disseminated encephalomyelitis (ADEM) and CIS) and diseases composed of multiple episodes of CNS demyelination (recurrent ADEM, MS, and NMO) [8]. Pediatric MS represents a particular MS subgroup with unique diagnostic challenges and many unanswered questions. It has long been an underrecognized and undertreated MS subgroup. While some aspects of the clinical disease in children resemble those of adults, children can also dramatically differ in clinical, radiological, and laboratory features. Pediatric neuromyelitis optica has a diverse clinical presentation and may be difficult to distinguish from multiple sclerosis in the early stages of the disease. To describe the spectrum of clinical phenotypes, laboratory and imaging features and treatment in pediatric patients with NMO are very important. Early diagnosis and immunosuppressive treatment may help slow the accumulation of severe disability. Children with MS and NMO may represent a particularly important group to study to gain a better understanding of their pathogenesis.

In this study, we aim to define the demographic, clinical, and laboratory features of patients affected from inflammatory demyelinating central nervous system diseases before the age of 18. 

## 2. Materials and Methods

### 2.1. Collection of Data

All data were collected from medical records of the patients who were being followed up at the Adult Neurology Department of Dokuz Eylül University Hospital. Patients, whose first acute inflammatory event of demyelination had started after the age of 18 were excluded from the study. Children that had been diagnosed with definite MS [5, 9] and NMO [7] before the age of 18 were eligible for the study. Patients who had been diagnosed at the age ≤18 and who were, at the time of study, over the age of 18, were also enrolled. 138 children were examined between 1968 and (March) 2012. Five of 138 cases were excluded due to lack of adequate medical records. Hence, 133 out of 2369 patients were included in the study. 63.2% (*n* = 84) of the patients were women, and 36.8% (*n* = 49) were men. The following data were analyzed on the basis of age at onset, sex, initial symptoms, course of disease, magnetic resonance imaging (MRI) features, presence of oligoclonal bands (OCBs), time of followup, treatment, and disability.

### 2.2. Magnetic Resonance Imaging (MRI)

MRIs, that was obtained within six months of the disease, was evaluated according to Barkhof-Tintore [9, 10] and KIDMUS criteria [11]. The diagnosis of NMO involved both clinical and imaging findings. At disease onset (first attack), a normal brain MRI, or lesions not meeting MS MRI criteria, fulfills one of the three major supportive diagnostic criteria for NMO [7]. 

### 2.3. The Neurological Impairment

Disability was measured by Expanded Disability Status Scale (EDSS) scores. EDSS score 3 represents mild-to-moderate disability without limitation in ambulation and 4 as moderate disability with an ability to walk at least 500 m unaided, score 6 walking with unilateral aid and score 7 wheelchair bound. We focused on score 4, that is, the walking ability of the patient is limited but they can walk without aid or resting for more than 500 m [12].

### 2.4. Statistical Analysis

All analyses were performed with Statistical Package For Social Sciences (SPSS version 15.0). Statistical significance was defined by an alpha level of 0.05. Descriptive statistics summarize clinical and demographic data. 

## 3. Results

### 3.1. Patient Characteristics (Table 1)

About 5.6% (133/2369) of the patients, with complete disease histories, experienced initial symptoms consistent with a demyelinating event under 18 years of age. The mean age at onset was 15.07 ± 2.72 (4–18), and it was 15.19 ± 2.78 (4–18) for females and 14.86 ± 2.62 (9–18) for males. The female/male ratio in the cohort was 1.7 : 1.

Of the patients, only 15 experienced onset below the age of 12 years and 118 experienced onset at and above the age of 12 years. The female/male ratio was 1.1 : 1 for under the age of 12. The youngest case was a 4-year-old girl with a polysymptomatic presentation after Mumps (ADEM-like onset). In 55 patients (41.4%), disease onset was before the age of 16, and in 78 patients it was after the age of 16 (58.6%). The female/male ratio was 1.3 : 1 for ages below 16, while it was 2.1 : 1 for ages above 16.

### 3.2. Onset of Disease

Patients experienced their first events with a myriad of neurologic symptoms including ON (19.4%), brainstem dysfunction (18.5%), sensory impairment (11.3%), ATM (10.5%), and motor dysfunction (10.5%) (Table 2). Polysymptomatic presentation—ADEM like the first demyelinating event (22.6%)—was the most common initial feature. All of these polysymptomatic cases experienced at least two non-ADEM attacks at some point in the course of their diseases.

### 3.3. Course of Disease

Of the 133 patients, 73.7% (*n* = 98) had RRMS, 15.8% (*n* = 21) SPMS, 6% (*n* = 8) CIS, 2.3% (*n* = 3) NMO, 1.5% (*n* = 2) Marburg, and 0.8% (*n* = 1) radiologically isolated syndrome (RIS). None of our cases experienced primary progressive (PP) MS course. And all our cases who presented like ADEM, a multifocal presentation with encephalopathy, were found to have MS in the course of their disease. Overall, 119 out of 2369 had MS: 5% of the total population in our center. Three patients meeting the inclusion criteria for NMO [7] were females. Their ages at the initial attack were six, nine, and fifteen, and their presenting symptoms were total ATM, unilateral ON, and unilateral ON, respectively. Tests for neuromyelitis optica immunoglobulin G (NMO-IgG) were positive for two patients. The only RIS patient was a 12-year-old boy with migraine. He had periventricular and callosomarginal gadolinium enhancing lesions in the brain, no lesion in the cervical spinal, and no OCBs. At the time of the study, he had been followed for two years and his last EDSS done within the last 6 months was 0.

### 3.4. Disease Duration, Treatment, and Prognosis

The mean disease duration of the demyelinating disease at the end of the follow-up period (which was March 2012 for the present study) was 9.48 ± 8.43 (range 1–44) years. At some point during the course of the disease, 68 cases (51.1%) received disease-modifying and immunosuppressant drugs: interferon beta (27.1%), glatiramer acetate (8.3%), and azathioprine (10.5%) (Table 3). The EDSS scores, which were recorded during the last visit within 6 months before the study, ranged from 0 to 9 with a median of 2.0 (2.22 ± 1.88) for 126 patients. The mean disease duration of the patients, whose EDSS score was ≤4, turned out to be 8.4 ± 7.7 (range 1–44, median 5) years, and whose EDSS score was >4, turned out to be 16.7 ± 9.0 (range 5–34, median 15) years.

### 3.5. MRI Features

MRI records of 111 patients were obtained. 97 patients had clinically definite multiple sclerosis. 11 MS patients (11.3%) did not initially present the diagnostic MRI features. All of the remaining multiple sclerosis patients fulfilled Barkhof-Tintore criteria (100%) and 88.7% fulfilled KIDMUS criteria. Cranial MRI of NMO patients was normal. For of those patients whose MRI did not meet both criteria, we employed Callen criteria [13]. Six of 11 (54.5%) patients' MRIs met the proposed MRI criteria for MS.

### 3.6. Cerebrospinal Fluid Oligoclonal Bands (CSF OCBs)

Among 133 patients, 35 OCB results were obtained from the medical records. OCBs were positive in 24 MS patients, one Marburg disease patient, and one CIS patient. OCB was negative in two of three NMO patients and in the RIS patient. 

### 3.7. Familial History

Six patients (4.6%) had a positive family history of MS. In five case, first-degree relatives and in one case, third-degree relatives had MS.

### 3.8. Comorbidities

The most frequent coexisting conditions were psychiatric disorders (depression and bipolar disorders) and migraine. Psychiatric disorders ascertained by Structured Clinical Interview for DSM-IV (SCID-I) [14] were seen in 5.2%, and the diagnosis of migraine based on International Headache Society criteria [15] was seen in 3.7% of the patients. Only two patients had type 1 diabetes mellitus, one of whom had a family history of MS in a first-degree relative.

### 3.9. The Group under the Age of 12

Fifteen patients experienced onset below the age of 12 years. Polysymptomatic presentation and brainstem involvement were the most common initial features, and the findings did not differ from patients above age 12 (*P* > 0.05). We obtained 10 of 15 patients MRIs. 5 MRIs were of MS patients, and all the MRIs met both Barkhof-Tintore and KIDMUS criteria. We reached the EDSS score of 14 of 15 cases, and of 85.7% had EDSS score of <4 with a median of eight years of disease duration. 

## 4. Discussion

In this study, 133 of 2369 (5.6%) ADD patients seen in our center experienced their first symptoms under 18 years of age: 15/2369 (0.63%) had an onset below the age of 12 years, 55/2369 (2.3%) below the age 16, and 78/2369 (3.3%) above the age 16. 

The most common initial event was polysymptomatic presentation in the present study. Some studies suggest that children are more commonly polysymptomatic (50–70%) than adults, although a monosymptomatic (30–50%) presentation is not uncommon [16–18].

Approximately 20% of the children ultimately diagnosed with MS were reported to demonstrate polysymptomatic features, encephalopathy, and widespread magnetic resonance imaging (MRI) lesions indistinguishable from ADEM [19]. On the other hand, none of our cases experienced encephalopathy and they had at least two non-ADEM attacks at some point in the course of their disease. ON and ATM were seen as initial presentation, respectively, in 19.4% and 10.5% of our patients, that were similar to the literature. ON and ATM were reported to be the initial presentation of MS in 14% to 35% and up to 10% of children, respectively [3, 16, 17, 19–23].

Childhood MS was found to be about 5% of our total patient population. This finding is consistent with several retrospective studies which have estimated the overall prevalence of MS, with onset before 18 years, to range from 1.6% to 10.5% of the total MS populations [3, 16, 20, 23–25]. There were only three early onset NMO patients in our study. In the literature, children may account for 10% of all NMO patients [26]. Only two children had Marburg variant; clinically characterized with rapid progression and an exceptionally severe course [27]. One of them died within one year from onset, and the second, in contrast to the cases in the literature, is still alive.

 More than half of our patients had been receiving immunomodulatory or immunosuppressive treatment at the time of enrollment. However, patients, who are still in childhood, constitute the minority of our treated patients. Prospective studies of immunomodulatory therapies to increase the recognition of the safety and tolerability of the agents [28–30] will encourage the physicians.

Although we did not apply a Kaplan Meier analysis for the time duration that passed to reach EDSS score 4 or greater, we found the median disease duration of patients, with EDSS score >4, to be 15 years. This result seems to be a little long, compared to that of a study in which 30 of 197 children (15%) documented EDSS score ≥4 after a median observation of 4.8 years (from second demyelinating attack) [31] and compared to that of another study that 13% of the children with MS experienced EDSS score ≥4 after a mean disease duration of only five years [19]. On the contrary, in a study that pediatric onset MS (<16 years) was found to be 10.5% of all MS patients, EDSS score of 4 was expected after 25 years of disease duration [15]. Also, in a prospective study in which 54 early onset (≤15) MS patients were present, EDSS scores greater than 4 were developed after a mean followup of 11 years [21].

Several diagnostic imaging criteria are being described and examined in pediatric multiple sclerosis. An increased risk of MS is associated with the presence of three out of four Barkhof criteria [9]; namely, one or more gadolinium-enhancing lesions, three or more periventricular lesions, one or more juxtacortical lesions, and one or more infratentorial or spinal cord lesions. The KIDMUS study [11] also identified MRI criteria that were associated with an increased risk of a second demyelinating event. These criteria were the presence of one or more lesions that were perpendicular to the long axis of the corpus callosum and the sole presence of well-defined lesions. Callen criteria for MS were established for a different purpose [13]. The findings of five or more T2 lesions, two or more periventricular lesions, and one or more brainstem lesions differentiated pediatric MS from other nondemyelinating neurological disorders. In our study MRIs of multiple sclerosis patients were evaluated according to Barkhof [9] and KIDMUS criteria [11]. All of the multiple sclerosis patients (*n* = 97) fulfilled Barkhof criteria (100%). Out of 97, 85 fulfilled KIDMUS criteria (88.7%). 12.6% of MS patients did not present the diagnostic MRI features initially; however in this group we found that 54.5% of them met the proposed Callen criteria, yet the criteria remain to be studied in larger pediatric cohorts. 

The number of patients, whose medical records of OCBs were achieved (*n* = 35), was low, but OCBs analyzed by isoelectric focusing were positive in 24 MS patients. OCBs were positive in 92% of 136 children studied in a German cohort, although CSF OCBs were not always in the CSF obtained at first attack [23, 32]. OCBs are rarely detected in patients with NMO [33, 34], and OCB was negative in two of our three NMO patients.

It is reported that an estimated 6 to 21% of children with MS have a first, second, or third-degree relative with MS [35–38]. In our study, 4.6% (*n* = 6) of the patients had a positive family history of MS in first-degree relatives (*n* = 5) and a third-degree relative (*n* = 1).

The most frequent coexisting conditions were psychiatric disorders (depression and bipolar disorders) (5.2%) in our study. Depression is a common comorbidity in adults with MS, estimated to affect 50–70% of adults at some point in the course of their diseases. Preliminary data from pediatric MS cohorts suggest that the rate of depression is similar, if not higher [39], using a structured psychiatric evaluation. Our frequency seems to be very low, that may be because the patients are less likely to seek help due to Turkey's cultural features which focus on physical disability and because the patients could not have the opportunity to be interviewed or to be screened for mood disorders in every visit. The frequency of migraine in the present study is consistent with some previous studies [40, 41]. Recent evidences suggest that demyelinating brainstem lesions might be among the factors responsible for the presence of migraine in MS patients [42, 43]. Familial susceptibility, young age, and female gender may predispose to both conditions or may be considered as additional risk factors [44]. Only one RIS patient was presented with migraine. The possibility of headache occurring as an MS onset symptom remains an open question. Headache is not generally considered as a symptom of MS, but, except for the types symptomatic of central lesions, its occurrence in patients without any other specific symptoms or signs of the disease, shown by some studies [45, 46], should be supported by a careful neuroradiological study and clinical monitoring [44]. 

## 5. Conclusions

All children presenting symptoms consistent with acute demyelination should be of particular interest as they require accurate evaluation aiming to decrease the burden of the disease when they reach adulthood. For this purpose, all features, such as demographic, immunologic, genetic, prognostic, and therapeutic, of early onset acute demyelinating diseases should be clarified, thus offering more specific treatment options.

## Figures and Tables

**Table 1:** Clinical and demographic features of the 133 patients.

Topic	
Female/male (*n*)	84/49 (1.7 : 1)
The mean age at onset (y)	15.07 ± 2.72 (4–18)
The mean disease duration (y)	9.48 ± 8.43 (1–44)
The mean EDSS score*	2.22 ± 1.88 (0–9)
Age at onset (*n*; f/m)	
<12 years	15 (1.1 : 1)
≥12 years	118 (1.8 : 1)
<16 years	55 (1.3 : 1)
>16 years	78 (2.1 : 1)
Course of disease (*n*; %)	
RRMS	98 (73.7%)
SPMS	21 (15.8%)
CIS	8 (6%)
NMO	3 (2.3%)
Marburg	2 (1.5%)
RIS	1 (0.8%)

y: years; f/m: female/male; RRMS: relapsing remitting multiple sclerosis; SPMS: secondary progressive multiple sclerosis; CIS: clinically isolated syndrome; NMO: neuromyelitis optica; RIS: radiologically isolated syndrome.

*The EDSS scores were recorded during the last visit within 6 months before the study for 126 patients.

**Table 2:** Onset of disease.

	*n*	%
Polysymptomatic	28	22.6
Optic neuritis	24	19.4
Brainstem dysfunction	23	18.5
Sensory impairment	14	11.3
Motor dysfunction	13	10.5
Transverse myelitis	13	10.5
Cerebellar dysfunction	6	4.8
Bilateral optic neuritis	2	1.6
Extrapyramidal symptoms	1	0.8

**Table 3:** Disease-modifying drugs and immunosuppressant drugs.

	*n*	%
IFN*β*-1a SC	22	16.5
Azathioprine	14	10.5
Glatiramer acetate	11	8.3
IFN*β*-1b	8	6
IFN*β*-1a IM	6	4.5
DMD + Mitoxantrone	2	1.5
Monthly IVMP + Mitoxantrone	1	0.8
Azathioprine + oral prednisolone	1	0.8
DMD + Azathioprine	1	0.8
Natalizumab	1	0.8
Laquinimod	1	0.8
Fingolimod	1	0.8

DMD: disease-modifying drugs; IVMP: intravenous methylprednisolone.
